# The Influence of Filler Loading and Alkaline Treatment on the Mechanical Properties of Palm Kernel Cake Filler Reinforced Epoxy Composites

**DOI:** 10.3390/polym14153063

**Published:** 2022-07-28

**Authors:** Tezara Cionita, Januar Parlaungan Siregar, Wong Ling Shing, Cheng Wan Hee, Deni Fajar Fitriyana, Jamiluddin Jaafar, Ramli Junid, Agustinus Purna Irawan, Agung Efriyo Hadi

**Affiliations:** 1 Faculty of Engineering and Quantity Surveying, INTI International University, Nilai 71800, Negeri Sembilan, Malaysia; 2 College of Engineering, Universiti Malaysia Pahang, Gambang 26300, Pahang, Malaysia; ramli@ump.edu.my; 3 Faculty of Health and Life Sciences, INTI International University, Nilai 71800, Negeri Sembilan, Malaysia; lingshing.wong@newinti.edu.my (W.L.S.); wanhee.cheng@newinti.edu.my (C.W.H.); 4 Department of Mechanical Engineering, Universitas Negeri Semarang, Kampus Sekaran, Gunungpati, Semarang 50229, Indonesia; deniifa89@mail.unnes.ac.id; 5 Faculty of Mechanical and Manufacturing Engineering, Universiti Tun Hussein Onn Malaysia, Parit Raja, Batu Pahat 86400, Johor, Malaysia; jamiluddin@uthm.edu.my; 6 Faculty of Engineering, Universitas Tarumanagara, Jakarta 11480, Indonesia; agustinus@untar.ac.id; 7 Mechanical Engineering Department, Faculty of Engineering, Universitas Malahayati, Jl. Pramuka No. 27, Kemiling, Bandar Lampung 35153, Indonesia; efriyo@malahayati.ac.id

**Keywords:** palm kernel cake, epoxy, filler, composites, surface modification

## Abstract

The manufacturing of materials, in conjunction with green technology, emphasises the need to employ renewable resources to ensure long-term sustainability. Re-exploring renewable elements that can be employed as reinforcing materials in polymer composites has been a major endeavour. The research goal is to determine how well palm kernel cake filler (PKCF) performs in reinforced epoxy composites. In this study, PKCF with 100 mesh was mixed with epoxy resin (ER) in various ratios ranging from 10% to 40% by weight. Hand lay-up with an open mould is proposed as a method for fabricating the specimen test. Surface modification of PKCF with varying concentrations of NaOH (5 wt.% and 10 wt.%) will be contrasted with the untreated samples. Using Fourier transform infrared spectroscopy (FTIR), thermogravimetric analysis (TGA), and differential scanning calorimetry (DSC), the effect of alkaline treatment will be examined. The tensile and maximum flexural strength of the untreated PKCF/ER composite were determined in this work, with a 30 wt.% of PKCF having the highest tensile strength of 31.20 MPa and the highest flexural strength of 39.70 MPa. The tensile and flexural strength were reduced to 22.90 MPa and 30.50 MPa, respectively, when the filler loading was raised to 40 wt.%. A 5 wt.% alkali treatment for 1 h improved the composites’ mechanical characteristics. Lastly, an alkali treatment can aid in the resolution of the problem of inadequate matrix and filler interaction. Alkaline treatment is a popular and effective method for reducing the hydroxyl group in fillers and, thus, improving interfacial bonding. Overall, palm kernel cake is a promising material used as a filler in polymer composites.

## 1. Introduction

Due to the poor biodegradability of synthetic fibre and the increasing environmental concern, researchers have been looking at natural fibre as a substitute for synthetic fibre in reinforced polymer composites, for developing ecologically acceptable home and industrial components, during the past few years [1]. Natural fibres, such as those obtained from agricultural wastes, are attracting interest in the polymer composite industry because of their many advantages, including being low cost and lightweight [2]. The utilisation of natural resources provides economic and environmental benefits obtained through waste recovery [3,4,5,6,7]. However, there are also drawbacks to using natural fibre in composites. The main issue observed, linked to the natural fibre-based polymer composite, is the counter characteristic of the polymer matrix and natural fibre, where the polymer matrix is a hydrophobic material. In contrast, natural fibre is a hydrophilic material [8]. This different characteristic makes the materials incompatible with each other. The incompatibility between the fibre and matrix composite produces a weak adhesion between them. Hence, it leads to the deterioration in the mechanical performance of the composite. According to past studies, there are numerous approaches for improving the composite’s mechanical performance, such as the inclusion of fibre treatment [9].

Consequently, to identify the solutions to this issue, researchers need to investigate the factors that influence the mechanical performance of the composite. per prior research, various aspects such as filler treatment, filler-loading composition, and polymer type have a significant impact on the composite’s mechanical performance [1,10,11]. As a result, given the previous investigation, this paper explores in-depth the potential composite elements that influence composite mechanical performance.

The composites’ tensile modulus and tensile strength are primarily determined by filler amount and size, filler properties, matrix interfacial bonding, void formation, moisture absorption by filler, and other factors, according to Arjmandi et al. (2015) [12]. According to Patel and Jain (2020), the decrease in tensile strength is because of the weak stress transfer between the filler material and matrix resin [13]. The majority of the previous authors looked at the influence of physical and chemical treatment, which is among the most well-known surface-modification approaches that help to promote matrix and fibre adhesion. This statement has been proven by several previous scholars, such as Olaitan et al. (2017), who fabricated epoxy-based composite incorporation of rice husk using treated fibre with 10% NaOH solution for 30 min [14]. To mention a few, Bisht and Gope (2018) developed a rice-husk flour/epoxy bio-composite and characterised the mechanical properties as well as the effect of alkali treatment on the composite [15]. In contrast, Emdadi et al. (2015) studied the impacts of chemical treatment on a rice-husk-reinforced composite [16]. The composite containing treated rice husk improved the adhesive qualities of composites, according to the researchers. Yeh et al. (2015) used a mixture of NaOH and maleic anhydride to treat a rice-husk-reinforced polypropylene composite. In conclusion, reinforcing material is a diffuse phase that usually consists of fibrous materials, including glass fibre and organic/natural fibre [17].

Oushabi et al. (2017) studied the alkaline-concentration impact in their investigation. They noted that 5% NaOH-treated date palm fibres resulted in an optimum tensile strength, which was 76% higher than raw fibres, and a 10% NaOH treatment resulted in a reduction in strength due to the surface damage of the fibres [18]. Gopinath et al. (2014) also concluded the same phenomenon for a jute-fibre-reinforced composite with a polyester matrix, where a 5% NaOH treatment of the fibres provided better mechanical properties compared to a 10% NaOH treatment [19]. The sisal–oil palm hybrid’s fibre mechanical properties, reinforced natural rubber composites with the effect of alkaline treatment, were investigated by John et al. (2008). It has been demonstrated that a 4% NaOH treatment produces a robust interface that improves fibre and rubber adhesion [20]. Lopattananon et al. (2006) observed the influence of surface treatment on the performance of a pineapple leaf fibre–natural rubber composite. The research work was done with various concentrations (1%, 3%, 5%, and 7%) of NaOH solution for the fibre treatment, and the treatment was done for 18 h with continuous stirring at room temperature. Among four concentrations, a 5% NaOH treatment maximised the composite tensile strength [21].

Ray et al. (2001) investigated the jute fibre composite’s flexural properties with different fibre loading (0, 8, 15, 23, 30, and 35 wt.%) and alkaline-treatment duration (0, 2, 4, 6, and 8 h). The composite properties showed optimum results with 4 h of treatment and 35 wt.% of fibre loading. The modulus has risen by 23% and the flexural strength by 20%, for 4 h of treatment [22]. Alkaline treatment, on the other hand, does not completely enhance the fibre or composite mechanical properties. For instance, Lopattananon et al. (2006) showed that the composite’s tensile strength was initially decreased for 1% and 3% NaOH fibre treatment [21]. Another instance is the effect of alkaline treatment on the mechanical characteristics of unsaturated polyester composites/sugar palm yarn with various fibre loadings, as reported by Norizan et al. (2018). A 1% NaOH solution was used for the alkaline treatment, which lasted 1 h. As a consequence, for both treated and untreated fibre, the flexural and tensile strength were optimum at 30 wt.% fibre loading [23]. However, among all the readings, the composite with treated fibre showed lower than the untreated fibre-reinforced composite. There are some parameters and conditions, such as treatment concentrations, duration, and suitability for the fibres or matrix [15,24,25]. Therefore, the right parameters of treatment for each type of fibre only can achieve improvements.

The objective of this study is to determine how filler loading and treatment affect the performance of palm kernel cake filler (PKCF) as a suitable reinforcing agent in epoxy composites.

## 2. Methodology

### 2.1. Materials

**Figure 1:**
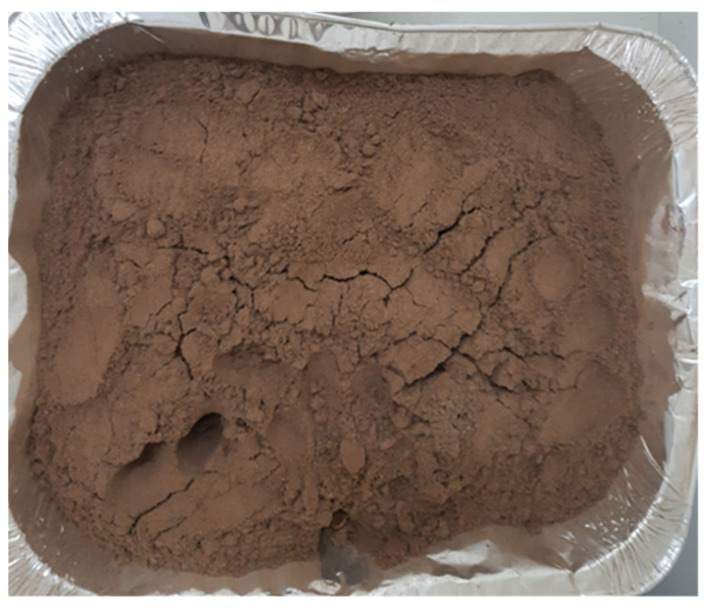
Palm kernel cake filler with size of 100 mesh.

The raw materials utilised in this research included palm kernel cake, sodium hydroxide or NaOH (C1143—HmbG), and epoxy resin (ER) Epikote 828 with epoxy hardener (651) product from Hexion, United States, which was purchased from the IZE solution company in Kuala Lumpur, Malaysia. The technical datasheet of epoxy resin as shown in Table 1. The Malaysian Palm Oil Board (MPOB) in Bangi, Selangor, Malaysia, provided the palm kernel cake. The density of palm kernel cake is 1.05 g/cm^3^. To remove residual oil, PKC was immersed and washed with distilled water before being air dried for 48 h. The palm kernel cake was then ground and sieved through a 100-mesh sieve. PKCF was dried in a vacuum oven at 105 °C for 24 h before being mixed with epoxy resin (Figure 1).

**Figure 2:**
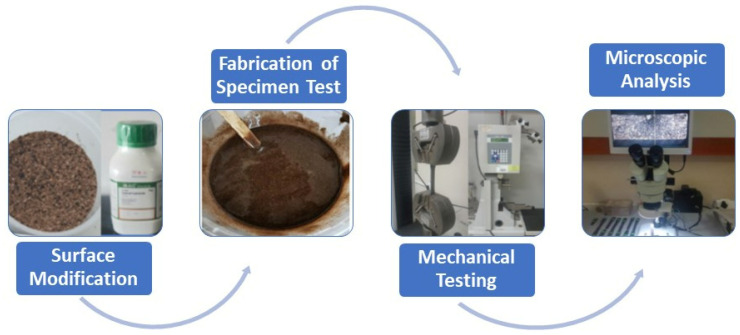
Research frameworks.

The methodology in this study was included; surface modification with alkaline treatment, mixing and fabrication of specimen tests, curing process, and mechanical test and analysis of fractured tensile specimen using Softop microscope. Figure 2 depicts the research methodology.

### 2.2. Fabrication of Specimen Test

Different concentration of filler loadings was prepared: 0%, 10%, 20%, 30%, and 40% (by weight) by mixing the UPKCF with ER. For each batch, the total weight of the mixture is maintained at 300 g. The weight of the filler, hardener, and epoxy is tabulated in Table 2. The hardener was slowly poured into the epoxy resin, and both were mixed in a container in a 3:1 ratio. A wooden stick was used to stir the matrix mixture slowly and uniformly. It was intended to prevent the formation of bubbles, but a few small bubbles remained inside. After 5 min, the filler was added in two to three turns to the matrix mixture. Thus, the composite mixture was constantly stirred for 5 min. Next, the composite mixture was put in a vacuum oven for 5 min at room temperature (25 °C) to eliminate air bubbles before pouring into the mould. Finally, the composite mixture was poured into the mould using the self-made funnel. The funnel is made of one-third of an A4 sheet of PVC paper, formed into a cone shape, ensuring that the mixture is evenly distributed in the mould. The composite was eventually cured for at least 24 h at room temperature. The specimens are characterised using the labels as shown in the last column.

### 2.3. Surface Modification with Alkaline Treatment

Table 3 shows the formulation parameter of surface modification of PKCF with alkaline treatment. Based on previous research findings [14,18], the 30 wt.% of untreated palm kernel cake filler (UPKCF) were treated for 1 h and 24 h with 5% and 10% NaOH solutions, respectively, to remove dirt and impurities from the filler surface. The alkali treatment transforms the filler surface into a rough surface, thereby enhancing the adhesion between the epoxy and filler surface [26]. The alkali treated fillers were rinsed with distilled water to neutralise the NaOH solution deposited on the filler surface, and then dried for 48 h at temperature 80 °C [27].

### 2.4. Mechanical Testing

**Figure 3:**
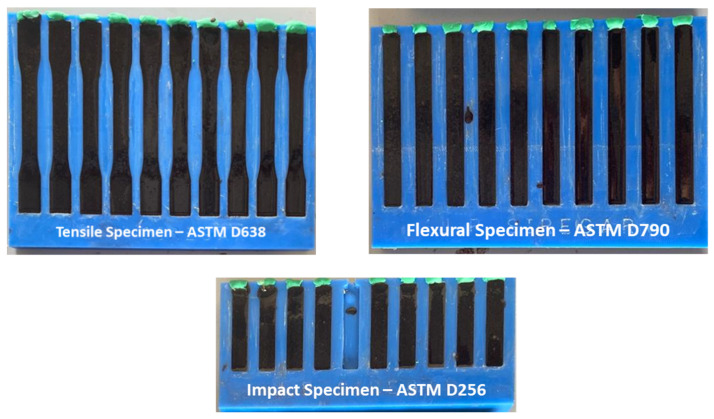
Specimens for tensile, flexural, and impact testing.

Figure 3 illustrates specimens for tensile, flexural, and impact testing conducted on treated and untreated PKCF/ER composites. Tensile properties, for instance, tensile modulus and tensile strength, are evaluated using PKCF/ER composite specimens in accordance with the American Society for Testing and Materials (ASTM D 638–04). An INSTRON 3369 universal testing machine is utilised to test a crosshead speed of 2 mm/min, and the average value is recorded. The modulus and flexural strength are evaluated using a three-point bending test on an INSTRON 3369 universal testing machine, which follows comparable tensile test techniques. Likewise, an Izod impact test composite measuring 127 mm × 12.7 mm × 3 mm was created in accordance with ASTM D256. The PKCF/ER composites’ mechanical properties will be studied between treated and untreated samples.

### 2.5. Microscopic Analysis

The tensile fractured specimens of untreated palm kernel cake filler (UPKCF) and treated palm kernel cake filler (TPKCF) of composite will be analysed under Meiji techno microscope.

### 2.6. Characterisation of PKFC/Epoxy Composite

The Nicolet iS50 Fourier transform infrared (FTIR) spectroscopy instrument from Thermo Fischer was utilised to analyse the potential chemical bonds present in both untreated and treated PKFF/ER composite samples. Based on the literature studies, the IR spectrometer is maintained between 4000 and 4000 cm^−1^ [28,29]. The thermogravimetric analyser of STA7200—Hitachi was used to study the thermal stability of untreated and treated filler composite. A powdered sample of 10 mg was put in an alumina crucible and kept in the furnace. The analysis was completed in a controlled environment where 20 mL/min of nitrogen gas was flowing. The rate of change of temperature was kept at 10 °C/min. The experiment was done from 30 °C to 800 °C. Differential scanning calorimetric (DSC) analysis for PKFC composites was performed using a Perkin Elmer DSC 8000. The nitrogen flow rate was 20 mL/min, and the heating rate was 10 °C/min for the test, which was carried out up to 350 °C above room temperature.

## 3. Results and Discussion

### 3.1. Tensile Properties

**Figure 4:**
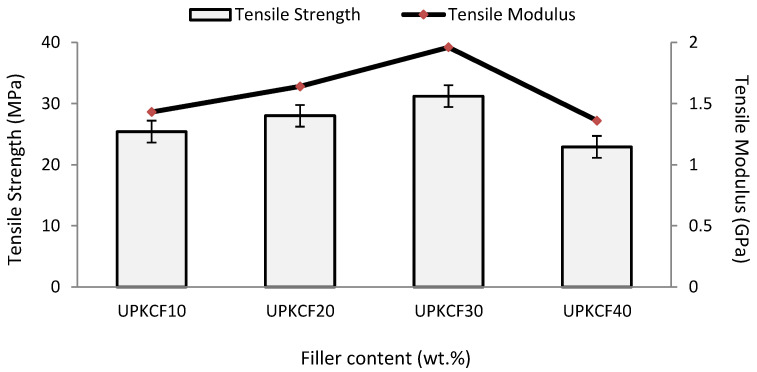
Tensile strength and tensile modulus of UPKCF/ER composites.

Figure 4 shows the untreated PKCF/ER composites’ tensile strength (TS) and tensile modulus (TM) at different filler loadings. The figure shows that increasing the amount of untreated palm kernel cake filler (UPKCF) from 10% to 30% raises the TM and TS from 0.95 GPa to 1.96 GPa and 25.40 MPa to 31.20 MPa, respectively. The rise in TM and TS reflects the findings study by Nagaraj et al. (2020). The researchers look into using date seed filler, in amounts varying from 5% to 50%, to reinforce vinyl ester composites. The results suggest that a 30% date seed filler concentration achieves the maximum tensile strength of 40.30 MPa [30]. This is due to the fact that the matrix’s tensile load distributed the created stress inside the filler. Therefore, the filler reinforcement carried the tensile load, limiting matrix fracture. Thus, it is more rigid and strong. With the increase in UPKCF to 40%, the tensile strength and tensile modulus dropped to 22.90 MPa and 1.36 GPa, respectively. At higher filler concentrations, this is attributable to poor bonding between the epoxy resin (ER) matrix and the filler. During tensile testing, it resulted in cavities and reduced strength [31].

**Figure 5:**
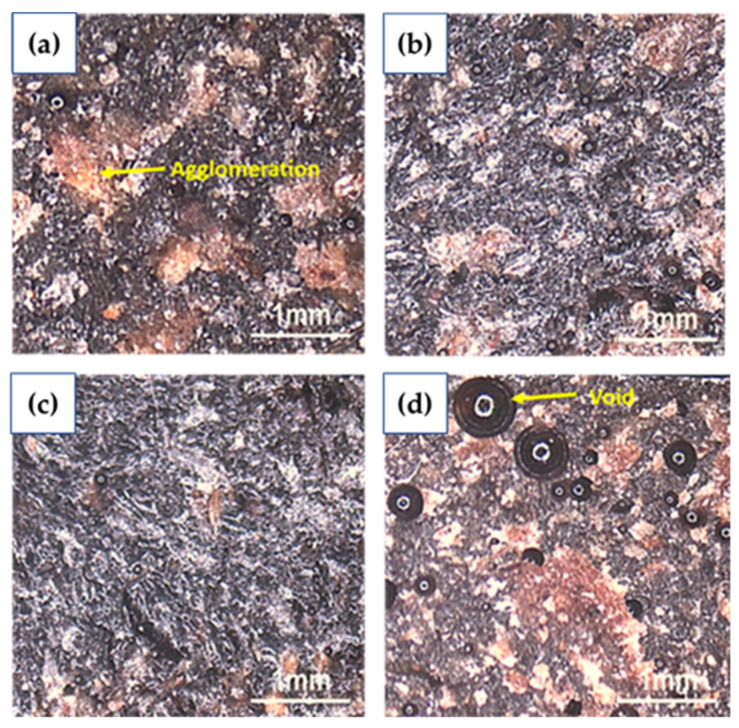
Microscopic of a tensile fractured UPKCF composites; (**a**) 10, (**b**) 20, (**c**) 30, and (**d**) 40 wt.%.

A Meiji Techno microscope was used to examine the surface tensile fractured of UPKCF/ER composites. Figure 5 depicts the surface morphology of ER containing 10%, 20%, 30%, and 40% UPKCF. Filler agglomeration was clearly visible at the fracture surface when loading 10%, 20%, 30%, and 40% fillers (Figure 5a–d). Increased filler-loading concentrations of up to 40% resulted in more agglomeration and voids in the surface of the composites. A similar result was stated by Khoshnoud et al. (2017). The reinforcements have a stronger tendency to agglomerate due to their high aspect ratio and low surface energy [32]. According to Kumar et al. (2018), the filler molecules agglomeration around the matrix, which precludes proper curing of the composites and results in a reduction in the flexural and tensile behaviour of the composites at a higher percentage of filler [33]. The ER’s low wetting capacity to the 40 wt.% filler in the composite in this study resulted in poor interfacial bonding between the filler reinforcement and the polymer epoxy matrix [34]. Figure 5c shows the surface morphology at 30 wt.% filler loading of UPKCF/ER composite, which showed good dispersion, less agglomeration, and fewer voids. This demonstrates that the quality of the filler–matrix mixture plays a crucial role in achieving excellent composite material properties [35]. Microscopic observation can illustrate the process of the filler concentration impact on UPKCF/ER composites mechanical properties, it can be concluded.

Table 4 summarises previous research findings on the tensile properties of specific filler-reinforced epoxy composites. Natural fillers have been employed as reinforcement agents for epoxy composites in a variety of ways, as shown in Table 4. The maximum tensile strength achieved in previous studies ranged from 15 MPa to 47.65 MPa, with the various loadings, sizes, and types of the filler [33,36,37,38,39,40]. In comparison, the findings of this study fall within the range (31.20 MPa) at 30 wt.% filler loading of UPKCF/ER. According to the comparison study, the tensile properties of the composite are affected by the filler type, filler size, and filler loading [41]. The filler materials’ distribution in the matrix has a major influence on composite performance [42].

### 3.2. Flexural Properties

**Figure 6:**
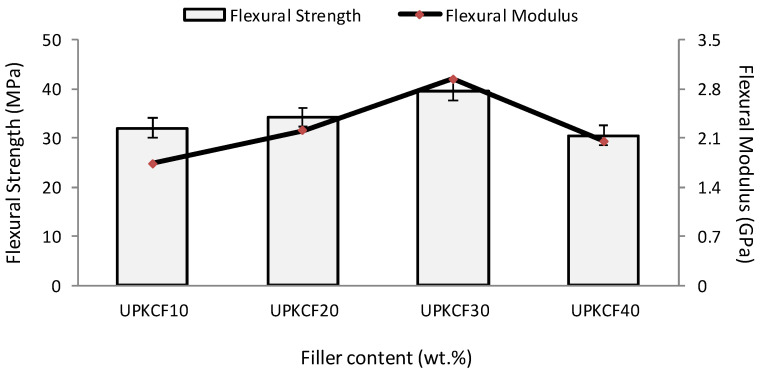
Flexural strength and flexural modulus of UPKCF/ER composites.

Figure 6 depicts the influence of filler loading on the flexural strength (FS) and flexural modulus (FM) of UPKCF-reinforced ER. Based on the findings, when the filler weight concentration increases, the maximum FS and FM values increase and reach a peak at 30% filler weight, before decreasing and reaching a minimum at 40% filler weight. With 40% filler loading, the UPKCF/ER composites decreased by 39% and 23% in FS and FM, respectively. This could be due to the increasing deterioration of interfacial bonding between the epoxy matrix polymer (hydrophobic) and UPKCF (hydrophilic). Depending on the adhesion of the fillers to the matrices, the introduction of fillers improved the flexural strength and flexural modulus [36]. The stiffness and interfacial area of contact of the composites were increased by adding fillers ranging from 10% to 30%, improving the flexural properties [45].

Table 5 gathers comparable results on the effect of filler concentration and size on the composites’ flexural properties from past research. From the table, the size of the fillers used to reinforce the polymer epoxy resin ranges from 2 µm to 30 mm. The optimal weight concentration of the filler used in the composite ranges from 10% to 50%. Several parameters, for instance, the filler size, the filler type, the filler concentration, and the interfacial adhesion between the fillers and the matrix, are thought to influence the composites’ flexural capabilities [33,36,37,38,40,43,44]. The highest flexural-strength value in this investigation is almost identical to that found in earlier experiments, ranging from 41 MPa to 53.4 MPa.

### 3.3. Izod Impact Properties

**Figure 7:**
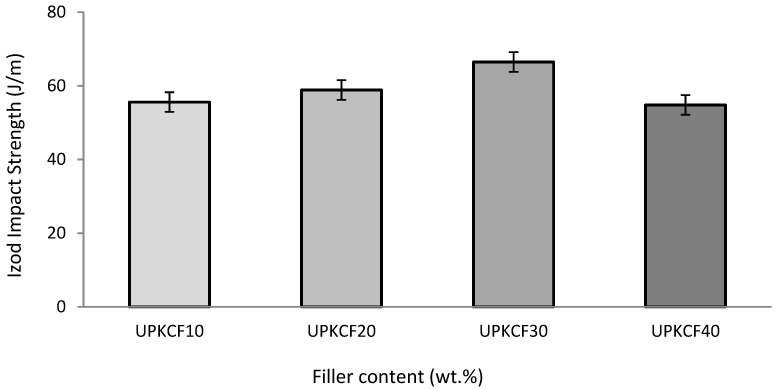
Izod impact strength of UPKCF/ER composites.

The Izod impact strength of PKCF/ER composites is shown in Figure 7. The tensile and flexural strength showed a similar pattern in this graph. At 30% filler loading, the composites’ maximal impact strength (66.48 J/m) is reached. As the filler loading rises, the impact strength starts increasing. The impact strength was reduced to 54.81 J/m at higher filler loading, resulting in the lowest impact strength compared to other concentration filler loadings. This could be because of the poor interfacial connection that exists between the filler and the matrix, in addition to the voids and agglomeration of the fillers that occur when there is a higher percentage of filler. It was also reported by Raju et al. [46] and Rizal et al. [47] that the impact strength of the composite specimens decreased with increasing filler loading. This behaviour of the composite specimens was observed to be similar.

Table 6 shows the results of past research on the Izod impact of various filler-reinforced epoxy composites. The types of filler and concentrations stated in the table have significantly improved the flexural and tensile properties of the composites described in the earlier portion, as shown in the table. The current study found that palm kernel cake filler (PKCF) at a concentration of 30% filler loading achieved the best mechanical properties, for instance, the flexural, tensile, and impact strength.

### 3.4. Effect of Alkaline Treatment

**Figure 8:**
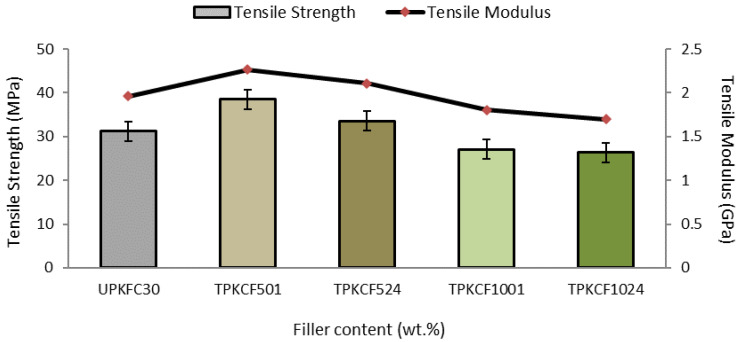
Effect of alkali treatment on PKCF/ER composites.

Figure 8 illustrates the alkaline-solution concentration influence on the tensile properties of PKCF/ER composites. The figure shows that for 30% filler loading (TPKCF501) treated with 5% NaOH solution in 1 h, both the tensile modulus and tensile strength greatly improved when contrasted with the untreated filler (UPKCF30). Treated filler with alkaline has improved the composites’ TS and TM by 23% and 15%, respectively. Compared with the soaking duration of 24 h, there are 7% and 7.7% improvements in TS and TM, respectively. When the fillers were soaked in sodium hydroxide solution with 10% NaOH concentration (TPKCF1001), the TS and TM worsened compared to the tensile properties of the untreated specimens. There was a 13.10% and 8.20% reduction in TS and TM, respectively. When the soaking time was extended to 24 h (TPKC1024), there were 2.60% and 6% reductions in TS and TM, respectively, compared to 1 h of soaking time.

**Figure 9:**
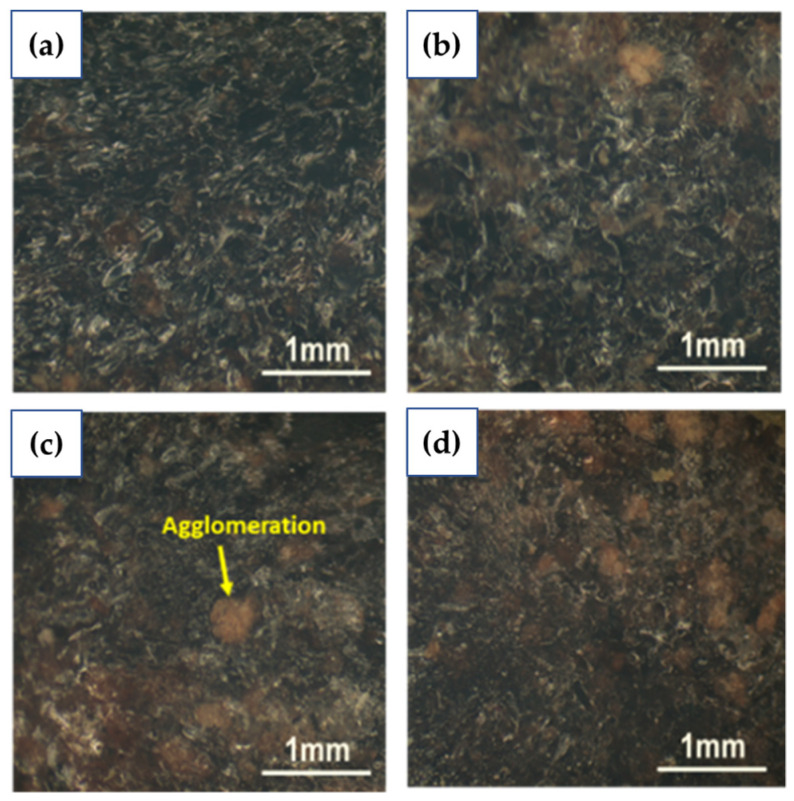
Microscopic of tensile-fractured TPKCF composites; (**a**) TPKCF501, (**b**) TPKCF524, (**c**) TPKCF1001, (**d**) TPKCF1024.

The results obtained from this study are along the lines of the earlier literature [49], which found the tensile properties are strongly influenced by the concentration of the alkaline. The interfacial bonding is enhanced by the build-up of a more effective area for mechanical interlocking between fibre and matrix due to fibrillation at a 5% NaOH solution concentration (Figure 9a,b). However, at 10% alkaline concentration with treatment duration of 1 h and 24 h (Figure 9c,d), the fibres are damaged and become brittle because of the high concentration of the alkali that is unsuitable for the treatment, as reported by Mahjoub et al. (2014) [50]. In addition, alkali treatment is used to change the surface of the filler from hydrophilic to hydrophobic by removing the components present on the surface. Finally, this approach is ideal for increasing the interfacial adhesion between the matrix and the fillers.

Table 7 summarises the similar findings from previous studies on the surface modification of natural fibre and filler using a sodium hydroxide solution. Table 6 shows that the range of alkaline treatment for natural filler is from 1% to 15%. The duration of the soak ranges from 1 to 24 h. The filler’s immersion time, for the best results, is recommended between 1 and 4 h. It has been revealed that treating various types of fibre and fillers with a 5% NaOH solution enhanced the composites’ tensile properties.

### 3.5. Fourier Transform Infrared Spectroscopy

**Figure 10:**
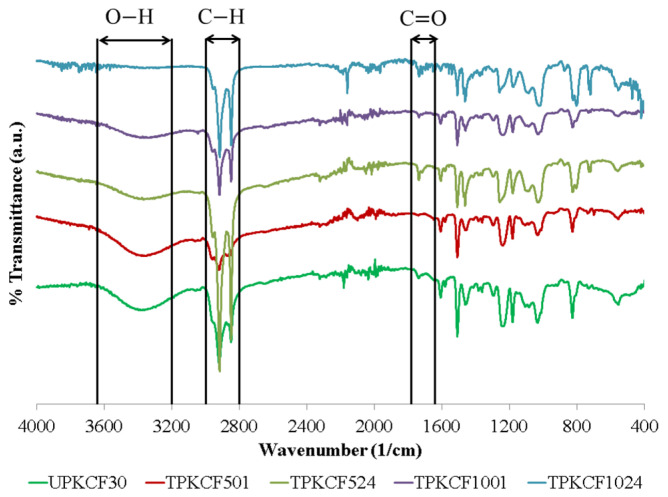
FTIR spectra of untreated and treated PKCF/ER composites.

The FTIR spectra of untreated and alkali-treated PKCF composites are shown in Figure 10. The UPKCF30 composites demonstrated a high absorption band between 3640 and 3200 cm^−1^ for the O–H stretching vibration of hydrogen-bonded cellulose. In contrast, the TPKCF composites exhibited no such significant absorption band. It shows that surface modification reduced the cellulose content. As a result of the OH absorption band, the UPKCF30 and TPKCF composites exhibited broad bands in the 3300 cm^−1^ regions. Due to the elimination of lignin and contaminants during alkali treatment, a corresponding drop in the intensity of all other peaks has also been seen in alkali-treated spectra [55,56,57]. The band appearing around 3000 and 2800 cm^−1^ corresponds to the C-H absorption band. The results for the treated palm kernel cake filler demonstrate the disappearance of the peak at approximately 1700 cm^−1^, confirming the elimination of lignin and hemicellulose following NaOH treatment. Meanwhile, the peaks detected at 1780 and 1640 cm^−1^ for untreated and treated palm kernel cake filler were attributable to the presence of C=O, which represents the groups in hemicellulose and lignin [56,57,58].

### 3.6. Thermogravimetric Analysis

**Figure 11:**
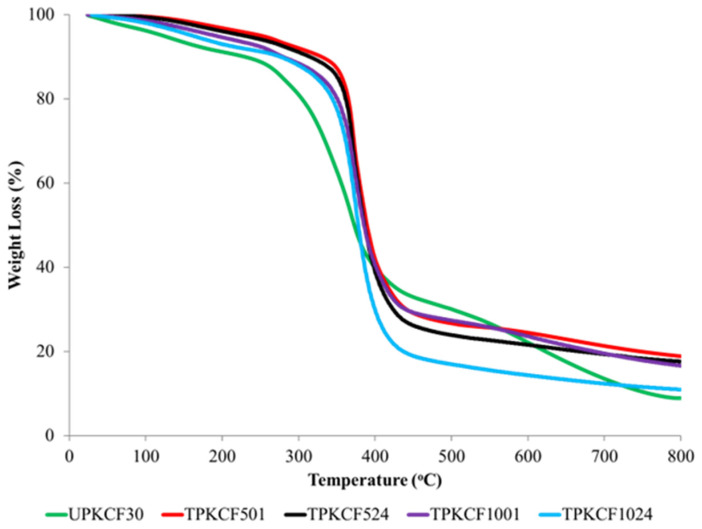
TGA curves of untreated and treated PKCF/ER composites.

**Figure 12:**
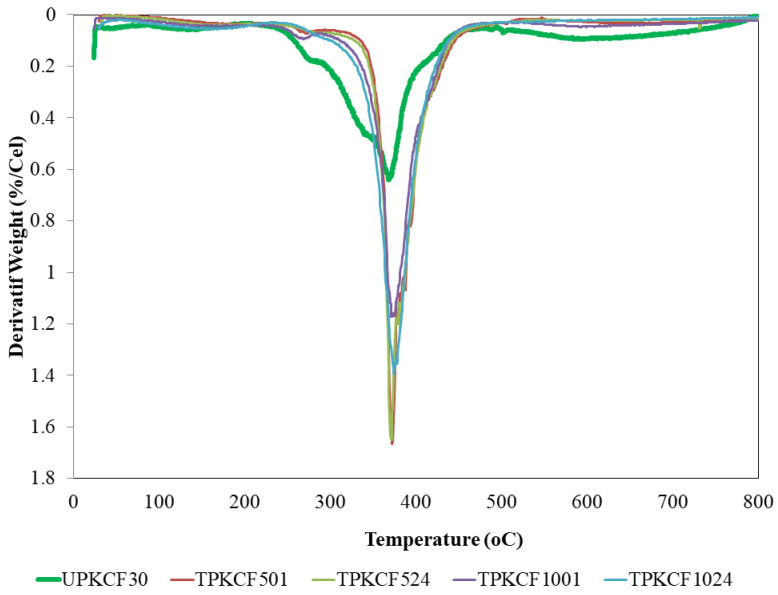
DTG curves of untreated and treated PKCF/ER composites.

TGA was used to study the thermal stability of untreated and alkali treated PKCF/ER composites. The TGA curves and their related thermogravimetric (DTG) derivative curves are depicted in Figure 11 and Figure 12, respectively. According to the TGA and DTG curves, both untreated and alkali treated samples displayed two separate stages of weight loss, at 30–150 °C and 200–600 °C, respectively [59]. The initial stage of weight loss was ascribed to the evaporation of water (30–150 °C). At temperatures of 105.4 °C, 122.9°C, 111.0 °C, 104.7 °C, and 103.7 °C, the initial stage of weight loss occurred in UPKCF30, TPKCF501, TPKCF524, TPKCF1001, and TPKCF1024, respectively. The degradation of polysaccharides, such as cellulose, hemicellulose, and lignin, was attributed to the second weight-loss stage, between 200 and 600 °C. Tmax is the degradation temperature that corresponds to the maximum weight loss and is related to the maximum decomposition temperature, which is also a crucial indicator of the thermal stability of the materials [58,59]. The maximum decomposition temperature of UPKCF30 was 313.6 °C. The maximum decomposition temperatures of TPKCF501, TPKCF524, TPKCF1001, and TPKCF1024, respectively, increased to 361.8 °C, 360 °C, 354.8 °C, and 349.7 °C, when PKCF was treated with 5% and 10% NaOH for 1 and 24 h, respectively. The degradation peak of treated PKCF occurred at higher temperatures than that of untreated PKCF, indicating that treated PKCF exhibited greater thermal stability at higher temperatures. With increasing concentrations of NaOH up to 5%, the maximum decomposition temperature increased to 361.8 °C and 360 °C for 1 and 24 h of treatment, respectively. However, when treated with a higher concentration of NaOH (10%), the highest decomposition temperature reduced to 354.8 °C and 349.7 °C for 1 and 24 h of treatment, respectively. It was determined that the thermal stability of PKCF might be enhanced by alkali treatment of the appropriate concentration and duration [59]. The findings of this investigation indicate that an alkaline treatment with a 5% NaOH concentration and a 1 h treatment time can yield composites with the greatest thermal stability compared to other specimens.

### 3.7. Differential Scanning Calorimetric

**Figure 13:**
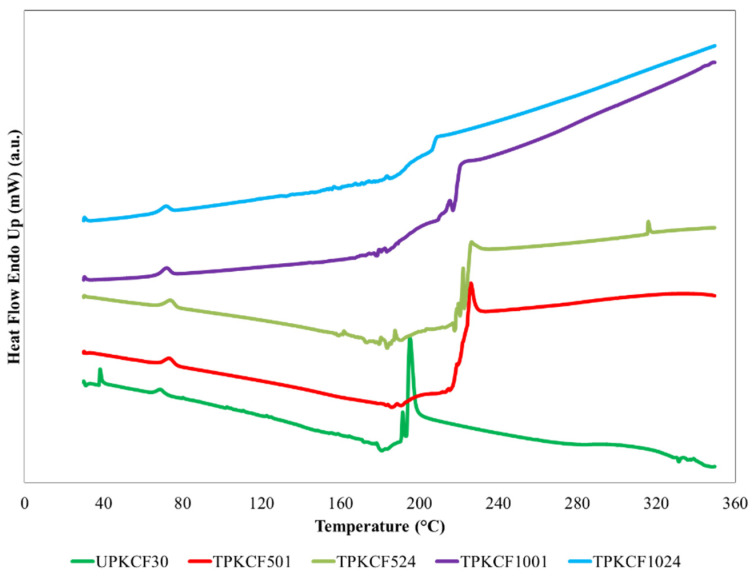
DSC thermogram of untreated and treated PKCF/ER composites.

Differential scanning calorimetric, often known as DSC, is a technique that measures the amount of energy that is either transferred to or removed from a sample as it is undergoing a change in its chemical or physical state [60]. The DSC curve for the PKCF/ER composite is shown in Figure 13. The thermal and chemical behaviour of the fibres was also observed on the DSC curve as the temperature increased. In PKCF/ER composites, endothermic peaks appear in the temperature range of 30–175 °C, indicating the presence of water molecule [58,61,62]. In this study, the presence of water molecules in UPKCF30 was shown by the presence of peaks at temperatures of 38.33 °C and 68.89 °C. On the other hand, in TPKCF501, TPKCF524, TPKCF1001, and TPKCF1024, the presence of water molecules is indicated by the presence of peaks at temperatures of 73.39 °C, 73.92 °C, 71.94 °C, and 71.59 °C, respectively. Alkaline treatment eliminated the peak that appeared at 38.33 °C. No exothermic or endothermic reactions were observed in the 80–160°C range, indicating that the PKCF is stable between these temperatures [61]. According to the literature data, the thermal decomposition of hemicellulose begins at about 180 °C and ends at about 350 °C [63]. As shown in Figure 12, UPKCF30 has two exothermic peaks, at 191.55 °C and 195.28 °C, on the DSC thermogram. This is due to the decomposition of hemicellulose. In the case of alkaline treatment of PKCF, hemicellulose peaks shifted to high temperatures of 226.05 °C, 226.26 °C, 220.84 °C, and 209.07 °C at TPKCF501, TPKCF524, TPKCF1001, and TPKCF1024, respectively. This is due to the removal of non-cellulosic materials such as hemicellulose and pectin. During alkaline treatment, the breaking of bonds present in the PKCF can have some effects and shift the peaks [64]. The appearance of these exothermic peaks after 250 °C confirmed the decomposition of cellulose in the PKCF at high temperatures [58,61,62,63,64].

## 4. Conclusions

The influence of filler loading and treatment on the mechanical properties of an epoxy composite reinforced with palm kernel cake filler (PKCF) has been investigated. For optimal tensile, flexural, and impact performance, the recommended concentration of untreated palm kernel cake filler (UPKCF) in an epoxy composite is 30 wt.% filler loading. When the filler concentration is increased to 40% by weight, the composite exhibits the lowest performance compared to other concentrations. The alkaline treatment with 5% NaOH and an hour of soaking time improved the composites’ performance in comparison to other alkaline concentrations. FTIR, TGA, and DSC were used to observe the characterisation of both untreated and treated PKCF/ER composites. The findings demonstrated that alkali treatment of PKCF enhanced the composites’ thermal stability. According to the findings of this study, PKCF should be exploited as a potential reinforcement element for polymer composites.

## Figures and Tables

**Table 1:** Technical datasheet of epoxy resin (Epikote 828).

Property	Value	Unit
Colour	100 max	Pt-Co
Epoxy group content	5260–5420	mmol/kg
Viscosity at 25 °C	12–14	Pa.s (Poise)
Density	1.16	Kg/L

**Table 2:** Composition of untreated PKCF/ER composites.

Filler Loading (%)	Filler Weight (g)	Epoxy Weight (g)	Hardener Weight (g)	Label
10	30	202.5	67.5	UPKCF10
20	60	180	60	UPKCF20
30	90	157.5	52.5	UPKCF30
40	120	135	45	UPKCF40

**Table 3:** Surface modification with alkaline treatment of PKCF/ER composites.

NaOH Concentration	Duration Time (h)	Label
0% (untreated)	0	UPKFC30
5%	1	TPKCF501
	24	TPKCF524
10%	1	TPKCF1001
	24	TPKCF1024

**Table 4:** Reported previous studies on tensile properties of various filler-reinforced epoxy composites.

Filler Type	Filler Size	Optimum Filler Loading (wt.%)	Remarks	Ref.
Banana	30 mm	20	Increased 37.31% compared to neat epoxy resin	[43]
Lagenaria Siceraria	7 mm	30	23.07 MPa	[36]
Rice husk	125 microns	-	Decreased compared to neat epoxy resin	[44]
Coconut shell	200–800 μm	20	30.60 MPa	[37]
Tea dust	-	50	15 MPa	[38]
Date palm	0.8–1 mm	50	25.76 MPa	[39]
Lantana camara	-	20	26.31 MPa	[40]
Wood dust	2 μm	10	47.65 MPa	[33]
Palm kernel cake filler	100 mesh	30	31.20 MPa	Current study

**Table 5:** Reported previous studies on flexural properties of various filler-reinforced epoxy composites.

Filler Type	Filler Size	Optimum Filler Loading (wt.%)	Remarks	Ref.
Banana	30 mm	20	Increased 10.13%	[43]
Lagenaria Siceraria	7 mm	30	48.40 MPa	[36]
Rice husk	125 microns	-	Decrease compared to neat epoxy resin	[44]
Tea dust	-	50	41 MPa	[38]
Lantana camara	-	20	53.4 MPa	[40]
Wood dust	2 microns	10	47.65 MPa	[33]
Palm kernel cake filler	100 mesh	30	39.70 MPa	Current study

**Table 6:** Reported previous studies on Izod impact of filler-reinforced epoxy composites.

Filler Type	Filler Size	Optimum Filler Loading (wt.%)	Remarks	Ref.
Banana	30 mm	20	Increased 80.99%	[43]
Lagenaria Siceraria	7 mm	30	0.75 J	[36]
Rice husk	125 microns	-	Decreased compared to neat epoxy resin	[44]
Tea dust	-	40	625 (J/m^2^)	[38]
Lantana camara	-	20	5.3 (J/cm^2^)	[40]
Hybrid pine needle fibre/pistachio shell filler	-	20 + 10	23.33 KJ/m^2^	[48]
Palm kernel cake filler	100 mesh	30	66.48 J/m	Current study

**Table 7:** Reported previous studies on alkali treatment of filler-reinforced epoxy composites.

Filler Type	Concentration of NaOH (%)	Duration (h)	Result (Optimum)	Ref.
Carnauba	1, 3, and 5	1, 2, and 3	5% at 1 h	[51]
Typha	5	1, 2, 4, and 8	5% at 4 h	[52]
Kenaf	5, 7, 10, and 15	1, 3, and 24	5% at 3 h	[53]
Carica Papaya bark	5	15, 30, 45, 60, 75, and 90 min	5% at 1 h	[24]
Raffia textilis	2.5, 5, and 10	12	5%	[54]
Palm kernel cake filler	5 and 10	1 and 24	5% at 1 h	Current study

## Data Availability

Data are contained within the article.
